# Evaluation of adverse reactions induced by anti-tuberculosis drugs among hospitalized patients in Wuhan, China: A retrospective study

**DOI:** 10.1097/MD.0000000000038273

**Published:** 2024-05-17

**Authors:** Cai Shi, Boning Yang, Jingxiang Yang, Wei Song, Ying Chen, Shuxiao Zhang, Hanyan Zhan, Yuanguo Xiong, Peipei Rong, Yi Luo, Jian Yang

**Affiliations:** a Department of Pharmacy, Renmin Hospital of Wuhan University, Wuhan, China; b Sydney Pharmacy School, Faculty of Medicine and Health, The University of Sydney, Sydney, NSW, Australia; c Department of Pharmacy, Wuhan Children’s Hospital, Tongji Medical College, Huazhong University of Science & Technology, Wuhan, China.

**Keywords:** adverse drug reactions, anti-tuberculosis drugs, incidence, risk factor

## Abstract

The study aims to estimate the incidence and risk factors of adverse drug reactions (ADRs) induced by anti-tuberculosis (TB) drugs. A single center retrospective analysis of patients taking anti-TB therapy from January 2016 to December 2018 in the hospital was conducted. Univariate and multivariate logistic regression analysis were used to identify these risk factors of ADRs induced by anti-TB drugs. Among 1430 patients receiving anti-TB therapy, 440 (30.77%) patients showed at least 1 ADR induced by anti-TB drugs. Hyperuricemia was the most common ADR, followed by hepatic function test abnormality, liver damage and gastrointestinal reactions. Significant differences (*P* < .05) were also seen in diabetes, age, treatment duration, type of TB (extrapulmonary) and some therapeutic regimens between ADR group and non-ADR group, respectively. Multivariate logistic regression analysis showed that treatment duration (OR = 1.029, 95%CI[1.018–1.040], *P* = .000), type of TB (extrapulmonary, OR = 1.487, 95%CI[1.134–1.952], *P* = .004) and some therapeutic regimens (HREZ, OR = 1.425, 95%CI[0.922–2.903], *P* = .001; HRZS, OR = 2.063, 95% CI[1.234–3.449], *P* = .006; HRZ, OR = 3.623, 95%CI[2.289–5.736], *P* = .000) were risk factors for ADRs induced by anti-TB drugs. Anti-TB drugs usually induced the occurrence of severe and frequent adverse effects, such as hyperuricemia. Treatment duration, HREZ, HRZS and HRZ regimens, and type of TB (extrapulmonary) should be considered as high-risk factors. Thus, it should be recommended to consider optimum management during anti-TB therapy, particularly hyperuricemia monitoring and hepatic function test.

## 1. Introduction

Globally, the best estimate was that 10.0 million people (range, 9.0–11.1 million) developed tuberculosis (TB) disease in 2017: 5.8 million men, 3.2 million women and 1.0 million children, but overall 90% were adults (aged ≥ 15 years).^[1]^ In 2017, the proportion of people who died from TB was 16%, down from 23% in 2000.^[1]^ TB, the leading cause of death from a single infectious agent, continued to threaten global health, accounting for 10 million cases and 1.2 million deaths worldwide in 2019.^[2,3]^ Thus, TB remains to be the major cause of significant morbidity and mortality worldwide. At present, China is still a high-burden country for TB, and the number of TB patients ranks second in the world.^[1]^ The prevalence of adverse drug reactions (ADRs) induced by anti-TB drugs has always been a matter of concern in China.

In order to control the TB epidemic, China National Tuberculosis Prevention and Control Scheme was established in 1990 for TB control, and directly observed treatment strategy therapy had been implemented since 1991, which was the cornerstone of the current strategy for TB control and covered the entire population of China.^[4,5]^ The regimen which required continually taking drug combinations of Isoniazid(H), Rifampicin(R), Pyrazinamide(Z), Ethambutol(E) and/or Streptomycin (S), was often used as the first-line therapy. However, some studies have shown that multi-combination therapy could cause severe ADRs, such as hepatotoxicity, gastrointestinal disorders, allergic reactions, arthralgia, neurological disorders and so on.^[6–8]^ According to a prospective study on 4304 TB in China, 649 patients (15.08%, 649/4304) had at least 1 ADR, and a total of 766 ADRs were identified.^[9]^ As to the overall prevalence of ADRs during anti-TB treatment, no consensus has been reached worldwide. For example, the overall prevalence of ADRs induced by anti-TB therapy ranged from 8.4% to 83.5% worldwide based on a systematic review.^[10]^

With the rapid development of current medical services in China, the prevalence of anti-TB-induced ADRs might have changed. At present, few studies investigated the prevalence of anti-TB-induced ADRs in Wuhan. Therefore, we planned to evaluate the incidence and risk factors of anti-TB-induced ADRs in the hospital for clinical prevention and treatment of ADRs.

## 2. Materials and methods

### 2.1. Study design and patients

The study was conducted with data from 1603 patients diagnosed with TB who received anti-TB treatment at Renmin Hospital of Wuhan University between January 2016 and December 2018. In total we recruited 1603 patients, of whom 1430 were enrolled in the study. The other 173 patients were excluded because of incomplete drug records. Of the 1403 patients, 918 were male and 512 were female. The mean age was 49.303 ± 19.52 years.

A pharmacist collected demographic data, medical and drug history, laboratory tests, reported ADRs, drug allergies, drinking, and smoking by using a designed data form. If an ADR induced by anti-TB drugs was detected, the form was filled in by a pharmacist and further confirmed by an infectious disease specialist. These data, including suspected drugs, the dosage, treatment duration, dechallenge and rechallenge results, were also recorded if applicable. Patients were divided into 2 groups ADR group and non-ADR group. The dosages of anti-TB drugs were: isoniazid (5 mg·kg^−1^ daily, maximum 300 mg daily), rifampicin (10 mg·kg^−1^ daily, maximum 1200 mg daily), pyrazinamide (15–30 mg·kg^−1^ daily, maximum 2000 mg daily), ethambutol (15 mg·kg^−1^ daily or 25–30 mg·kg^−1^ daily, maximum 2500 mg daily) and streptomycin (20–30 mg·kg^−1^ daily, maximum 750 mg daily). Drug administration and therapeutic schedule were adjusted according to patient age, medical condition and the combination regimens by the attending physician.

### 2.2. ADR definition

An ADR was defined as “an unintended and harmful reaction to a medication, and which occurs at doses normally used in humans.” Serious ADRs were defined as “any untoward medical occurrence that at any dose results in death, requires hospital admission or prolongation of existing hospital stay, results in persistent or significant disability/incapacity, or is life-threatening.” The causality of ADR was analyzed by using the WHO Uppsala Monitoring Centre criteria. ADR reports with a certain, probable or possible causality assessment were included in this study. Observed ADRs were classified into 3 categories mild, moderate, and severe. Liver damage was defined as an increase in serum alanine aminotransferase, aspartate transaminase, alkaline phosphatase and total bilirubin level at the same time and one of the indicators higher than twice the upper limit of normal (ULN). Hepatic function test abnormality was accepted as an increase in serum alanine aminotransferase, aspartate transaminase, alkaline phosphatase and total bilirubin level within 2 × ULN. Hyperuricemia was defined as an increase in uric acid levels of more than 8 mg/dL. Anemia was described as hemoglobin concentration < 10g/dL in female and < 11g/dL in male inpatients without a history of anemia or more than 1 g/dL drop in hemoglobin concentration after anti-TB treatment. Neutropenia and thrombocytopenia were recognized as a drop in absolute neutrophil count and platelet count equal to or <1500 cells/mm^3^ and <150,000 cell/mm^3^, respectively. Except liver dysfunction, hematologic system disorders and renal impairment were identified by laboratory test, other ADRs including allergic reactions, arthralgia and nervous system disorders were identified based on symptoms. Nervous system disorders included auditory nerve damage, optic nerve damage, peripheral nervous damage and central nervous system damage. “Others” referred to those ADRs could not classified to above types, such as hyperthyroidism, interstitial pneumonia, lipsotrichia and so on.^[9]^

### 2.3. Statistical methods

The SPSS 24.0 for Windows (IBM Corp., Armonk, NY) were used to perform the statistical analyses. Continuous variables were expressed as mean ± SD or median (range). Categorical variables were presented as frequencies and percentages. Univariate and multivariate logistic regression analysis were used to identify the risk factors of ADRs. A bilateral *P* value <.05 was considered statistically significant.^[11]^

### 2.4. Ethical approval

The Renmin Hospital Ethics Committee of Wuhan University approved the retrospective study (protocol number: 2018KC124). Owing to the retrospective nature of the study with no interventions performed, the informed consent was waived.

## 3. Results

### 3.1. Characteristics of TB patients with ADRs

440 patients experienced at least 1 ADR (30.77%, 440/1430), including 300 patients with 1 ADR, 102 with 2 ADRs, 23 with 3 ADRs and 14 with 4 ADRs and 1 with 5 ADRs. Thus, a total of 634 ADRs were detected. As shown in Table 1, the most frequent system-organ classes affected by ADRs were metabolic and nutritional disorders (29.02%,184/634), liver and biliary system disorders (28.55%, 181/634) and gastrointestinal system disorders (11.52%, 73/634). Hyperuricemia (27.92%, 177/634), hepatic function abnormality (17.03%, 108/634), liver damage (10.88%, 69/634), nausea and vomiting (6.62%, 42/634), were top-four ADRs in the study. Of 634 ADRs, 35 ADRs (5.52%, 35/634) were identified as certain, 461 ADRs (72.71%, 461/ 634) as probable, and 138 ADRs (21.77%,138/634) as possible.

**Table 1:** Frequency of system-organ classes involved in ADRs induced by anti-TB drugs and their major clinical manifestations.

Site of reactions	Frequency(n)	Rate(%)	ADR(n)
Liver and biliary system disorders	181	28.55	Liver damage(69), hepatic function test abnormality (108), Jaundice(2), upper abdominal pain(2)
Urinary system disorders	35	5.52	Kidney damage(1), hematuria(30), elevated creatine kinase(3), dysuria(1)
Gastrointestinal system disorders	73	11.52	Nausea and vomiting(42), stomach pain (10), abdominal pain(10), diarrhea(4), bloating(4), poor appetite(3)
Metabolic and nutritional disorders	184	29.02	Hyperuricemia(177), edema(3), elevated blood sugar (4)
Skin and its attachments or flu-like illness	16	2.52	Rash and itching(13), increased rash(1), delayed allergy(2)
Musculoskeletal system damage	7	1.10	Bilateral thigh muscle pain(1), gout(5), lumbago(1)
Systemic injury	18	2.84	Self-sweat(3), drug fever(14), limb weakness(1)
Visual and hearing impairment	22	3.47	Ear canal pain(3), hearing decrease (6), blurred vision(8), dry eyes(3), conjunctival hyperemia(1), olfactory loss(1)
Central and peripheral nervous system disorders	35	5.52	Weakness(5), poor sleep(3), lethargy(3), numbness of the limbs (3), involuntary sway of the upper limbs (7), dizziness and headache (11), secondary epilepsy (1), memory loss(1), unstable walking(1)
Blood system disorder	52	8.20	Leucocytopenia(15), thrombocytopenia(2), neutrophilopenia(13), anemia (16), erythropenia (6)
Others	11	1.74	Cough and sore throat(3), palpitation(1), chest tightness(1), sternal discomfort(1), rifampicin resistance(4), photosensitive reaction(1)
Total	634	100.00	

ADR = adverse drug reaction, ADRs = adverse drug reactions, TB = tuberculosis.

The median (inter-quartile range) onset of the ADRs was 8.43 ± 7.31 days (1–90 days). Among 634 ADRs, the earliest ADR occurred was stomach upset, hematuria at 1 day, and the latest ADR was liver damage in the continuous use of isoniazid, rifampicin and ethambutol triple anti-TB treatment after 90 days. 326 ADRs (51.42%, 326/634) were detected within 7 days after initiation of anti-TB treatment, 532 ADRs (83.91%, 532/634) within 15 days.

Among 634 ADRs, 415 ADRs (65.46%, 415/634) were mild, 130 ADRs (20.50%, 130/634) were moderate, and 89 ADRs (14.04%, 89/634) were severe. With regards to outcome of ADRs, 47 ADRs (7.41%, 47/634) were cured, 373 ADRs (58.83%, 373/634) were improved, 179 ADRs (28.23%, 179/634) were not known, 35 ADRs (5.52%, 35/634) were worsen, and no death.

The main treatment regimens and the frequency of their associating ADRs were: HRE (32.33%, 205/634), HREZ (29.34%, 186/634), HRZS (14.98%, 95/634), HRZ (12.46%, 79/634), HE (2.05%, 13/634), HRES (1.89%, 12/634), HEZ (1.89%, 12/634), RZ (0.95%, 6/634), and HR (0.79%, 5/634), respectively (shown in Table 2).

**Table 2:** Frequency of ADRs induced by different anti-TB regimens.

Regmens	Frequency(%)
E	0 (0)
H	3 (0.47)
HE	13 (2.05)
HEZ	12 (1.89)
HEZS	0 (0)
HES	2 (0.32)
HZ	2 (0.32)
HZE	0 (0)
HR	5 (0.79)
HRE	205 (32.33)
HREZ	186 (29.34)
HREZS	3 (0.47)
HRES	12 (1.89)
HRZ	79 (12.46)
HRZS	95 (14.98)
HRS	3 (0.47)
Z	1 (0.16)
ZS	0 (0)
R	3 (0.47)
RE	0 (0)
REZ	1 (0.16)
REZS	2 (0.32)
RZ	6 (0.95)
RZS	1 (0.16)
Total	634 (100)

ADRs = adverse drug reactions, E = ethambutol, H = isoniazid, R = rifampin, S = Streptomycin, TB = tuberculosis, Z = pyrazinamide.

### 3.2. Risk factors for ADRs

Table 3 summarized the clinical and demographic variables between non-ADR group (n = 990) and ADR group (n = 440). As shown in Table 4, the univariate analysis revealed that there were no significant differences in gender, smoke, drink, drug allergy or TB treatment history between ADR group and non-ADR group. From the perspective of history of diseases, diabetes (OR = 0.642, 95%CI [0.428–0.964], *P* = .032) could influence the incidence of ADRs, while hepatitis B virus (HBV), nephropathy, hypertension and nonalcoholic fatty liver disease (NAFLD) seemed to have no influence on the incidence of ADR. However, age (OR = 0.956, 95%CI [0.9564–1.363], *P* = 0008), treatment duration (OR = 1.368, 95% CI [1.106–1.576], *P* = .000), type of TB (extrapulmonary, OR = 1.603, 95% CI [1.244–2.064], *P* = .000), HREZ (OR = 5.645, 95%CI [1.929–16.521], *P* = .002), HRZS(OR = 10.244, 95%CI[3.341–31.408], *P* < .001 and HRZ(OR = 4.078, 95% CI [1.364–12.188], *P* = .012) appeared to be related to the incidence of ADRs during anti-TB therapy.

**Table 3:** Summary of ADR group and non-ADR group.

Variable	Non-ADR group n = 990(69.23%)	ADR group n = 440(30.77%)
Age (IQR), yr	51 (3–94)	47 (0–96)
Gender, n(%)		
Male	638 (64.44)	280 (63.64)
Female	352 (35.56)	160 (36.36)
Treatment duration, d	17	14
History of disease		
HBV, n(%)		
No	867 (87.58)	399 (90.68)
Yes	123 (12.42)	41 (9.32)
Nephropathy, n(%)		
No	906 (91.52)	410 (93.18)
Yes	84 (8.48)	30 (6.82)
Hypertension, n(%)		
No	806 (81.41)	369 (83.86)
Yes	184 (18.59)	71 (16.14)
Diabetes, n(%)		
No	879 (88.79)	407 (92.50)
Yes	111 (11.21)	33 (7.50)
NAFLDs, n(%)		
No	957 (96.67)	430 (97.73)
Yes	33 (3.33)	10 (2.27)
History of drug allergy, n(%)		
No	905 (91.41)	399 (90.68)
Yes	85 (8.59)	41 (9.32)
Smoke, n(%)		
No	750 (75.76)	333 (75.68)
Yes	240 (24.24)	107 (24.32)
Drink, n(%)		
No	864 (87.27)	388 (88.18)
Yes	126 (12.73)	52 (11.82)
Tuberculosis treatment history, n(%)		
Primary treatment	797 (80.51)	365 (82.95)
Re-treatment	193 (19.49)	75 (17.05)
Type of tuberculosis, n(%)		
Pulmonary	342 (34.55)	109 (24.77)
Extrapulmonary	648 (65.45)	331 (75.23)
Therapeutic regimens, n(%)		
HR	28 (2.83)	4 (0.91)
HRE	494 (49.90)	146 (33.18)
HREZ	155 (15.66)	125 (28.41)
HRZ	103 (10.40)	60 (13.64)
HRZS	41 (4.14)	60 (13.64)

ADR = adverse drug reaction, E = ethambutol, H = isoniazid, HBV = hepatitis B virus, IQR = interquartile range, NAFLDs = nonalcoholic fatty liver disease, R = rifampin, S = Streptomycin, Z = pyrazinamide.

**Table 4:** Logistic regression analysis of independent risk factors for ADRs.

Variable	Univariate analysis		Multivariate analysis*	
	OR (95% CI)	*P* value	OR (95% CI)	*P* value
Age	0.956 (0.9564–1.363)	.008	0.996 (0.989–1.002)	.204
Gender				
Female	Ref			
Male	0.996 (0.764–1.220)	.769		
Treatment duration	1.368 (1.106–1.576)	.000	1.029 (1.018–1.040)	.000
History of disease				
HBV				
No	Ref			
Yes	0.724 (0.499–1.052)	.090		
Nephropathy				
No	Ref			
Yes	0.789 (0.512–1.217)	.284		
Hypertension				
No	Ref			
Yes	0.843 (0.624–1.138)	.264		
Diabetes				
No	Ref			
Yes	0.642 (0.428–0.964)	.032	1.333 (0.859–2.069)	.200
NAFLDs				
No	Ref			
Yes	0.674 (0.329–1.381)	.281		
History of drug allergy				
No	Ref			
Yes	1.094 (0.740–1.617)	.652		
Smoke				
No	Ref			
Yes	1.004 (0.773–1.305)	.975		
Drink				
No	Ref			
Yes	0.919 (0.651–1.297)	.631		
Tuberculosis treatment history				
Primary treatment	Ref			
Re-treatment	0.849 (0.632–1.139)	.274		
Type of tuberculosis				
Pulmonary	Ref			
Extrapulmonary	1.603 (1.244–2.064)	.000	1.487 (1.134–1.952)	.004
Therapeutic regimens				
HR	Ref			
HRE	2.069 (0.714–5.994)	.180		
HREZ	5.645 (1.929–16.521)	.002	1.425 (0.922–2.903)	.001
HRZ	4.078 (1.364–12.188)	.012	3.623 (2.289–5.736)	.000
HRZS	10.244 (3.341–31.408)	<.001	2.063 (1.234–3.449)	.006

ADRs = adverse drug reactions, CI = confidence interval, E = ethambutol, H = isoniazid, HBV = hepatitis B virus, NAFLDs = nonalcoholic fatty liver disease, OR = odds ratio, R = rifampin, S = Streptomycin, Z = pyrazinamide.

* Values are adjusted for age, treatment duration, diabetes, type of tuberculosis (extrapulmonary), HREZ, HRZ, HRZS.

From Table 4, only type of TB (extrapulmonary), treatment duration and some therapeutic regimens were independently associated with ADRs during anti-TB therapy, respectively. Therefore, treatment duration (OR = 1.029, 95% CI [1.018–1.040], *P* = .000) and type of TB (extrapulmonary, OR = 1.487, 95% CI[1.134–1.952], *P* = .004) was more likely to develop ADRs during anti-TB therapy. On the other hand, among these combination regimens of drugs, HREZ(OR = 1.425, 95%CI[0.922–2.903], *P* = .001), HRZS(OR = 2.063, 95%CI[1.234–3.449], *P* = .006) and HRZ(OR = 3.623, 95% CI [2.289–5.736], *P* = .000) were also risk factors for ADRs during anti-TB therapy.

## 4. Discussion

The ADRs associated with anti-TB drugs, particularly first-line anti-TB drugs, have been reported widely. The overall prevalence of ADRs induced by anti-TB therapy ranged from 8.4% to 83.5% worldwide.^[10]^ In China, the overall incidence of anti-TB drugs induced ADR of 117 studies was 12.62% from 1996 to 2005.^[12]^ In this study, the incidence of ADRs was 30.77% during anti-TB therapy, which was higher than those in previous studies in China.^[9,12,13]^ Moreover, some studies showed that in China, liver dysfunction or gastrointestinal reactions were the most common ADR.^[9,12,13]^ For example, Xiaozhen Lv et al found that liver dysfunction occurred most frequently (273, 6.34%), of which 106 experienced liver toxicity (2.55%) in a prospective study of 4304 TB patients in China.^[9]^ Tao Zhang et al also reported that 462 (462/2091, 22.1%) Chinese patients developed ADRs, with liver injury and gastrointestinal reactions constituting the most common ADRs. Specifically, 9.8% and 6.3% developed liver injuries and gastrointestinal reactions, respectively.^[13]^ In mainland China, Xia et al reported an incidence of liver injure (11.9%), which was the highest among all kinds of ADRs induced by anti-TB therapy by reviewing the reports published from 1996 to 2005.^[4]^ However, in the study, hyperuricemia (12.38%,177/1430) was the most common ADR, followed by hepatic function test abnormality (7.55%,108/1430), liver damage (4.83%, 69/1430), and gastrointestinal reactions (2.94%, 42/1430). Therefore, our results were different from previous studies.^[9,12,13]^ The variation in the prevalence of ADRs might be attributed to the differences in patients’ characteristics, study design, study population, therapy regimens, region and the definition criteria of ADRs.

In the study, the median onset of ADRs was 8.43 ± 7.31 days (range, 1–90 days). This finding differed from other study, which reported that the mean onset of ADR after starting the anti-TB treatment regimen was 56.40 ± 58.29 days (range, 1–180 days).^[14]^ This difference might be due to treatment schemes, study design and the shorter period of hospitalization. In the study, 317 (50%, 317/634) ADRs were detected in 7 days after initiation of anti-TB treatment, suggesting that all patients should be closely monitored for ADRs during the first week of treatment in order to identify severe ADRs and apply appropriate intervention in time.

ADRs were multifactorial^[15,16]^ and the major determinants were unadjusted prescribed doses of medications, patient age, nutritional status, alcohol consumption, altered liver and kidney function and human immunodeficiency virus infection.^[17,18]^ Old age and being male were considered as risk factors for developing ADR induced by anti-TB drugs, while smoking and a relatively long treatment phase was also known as risk factors for hepatotoxicity induced by anti-TB drugs.^[19]^ Studies also have identified some factors contributing to ADR-related hospitalization, including older age, female gender, increased number of co-morbidities, increased number of medications, renal diseases, liver diseases, history of ADRs and so on.^[20]^

Interestingly, in the study, gender, smoke, drink, HBV, NAFLDs, TB treatment history, and history of drug allergy had no significant association with the risk of ADRs, respectively. Age, diabetes, treatment duration, type of TB (extrapulmonary) and some therapeutic regimens were associated with the occurrence of ADRs, respectively.

Old age was one of the risk factors involved in the development of ADRs induced by anti-TB drugs in several previous studies.^[21–23]^ On the contrary, Tao Zhang et al reported that age did not increase the risk of ADRs for elders during anti-TB treatment. Compared with the 19–39 year old group, age over 60 years was a protective factor for liver injury.^[13]^ In the study, age exhibited a significant difference between ADR group and non-ADR group(*P* = .008). For instance, the median age of ADR group was 47 years, while median age of non-ADR group was 51 years, meaning that younger patients could increase the occurrence of ADRs than older patients. However, the subsequent multivariate analysis revealed that age might not be a risk factor for ADRs, which was similar to the study.^[13]^

Patients with preexisting liver diseases were more likely to develop ADRs.^[20]^ Interestingly, HBV and NAFLDs were not risk factors for ADRs in the study. To date, it was also seldom reported whether diabetes could influence the incidence of ADRs during anti-TB therapy. However, diabetes displayed a significant difference between ADR group and non-ADR group in this study. But the incidence of ADRs in patients without diabetes (31.65 %, 407/1286) was slightly higher than that in patients with diabetes (22.92%, 33/144), which suggested that diabetes might not increase the occurrence of ADRs.

In the study, the median treatment duration of ADR group was 14 days, while median treatment duration of non-ADR group was 17 days. The regression analysis showed that treatment duration (OR = 1.029, 95%CI [1.018–1.040], *P* = .000) might be the risk factor for ADRs, implying that shorter treatment duration could increase the occurrence of ADRs.

Although previous studies^[9,12,13]^ have evaluated ADRs during anti-TB therapy, those studies did not consider these differences among treatment regimens. From Table 4, we found that some treatment regimens were significant difference between non-ADR and ADR groups. Notably, of 1430 patients receiving anti-TB drugs, 640 patients were treated by HRE(22.81%, 146/640), and its incidence of ADRs was lower than those of the treatment regimens from 3 to 4 drugs, such as HREZ(44.64%,125/280), HRZS(59.41%, 60/101), and HRZ (36.81%, 60/163). Among these therapeutic regimens, HREZ (OR = 1.425, 95%CI [0.922–2.903], *P* = .001), HRZS (OR = 2.063, 95%CI [1.234–3.449], *P* = .006), and HRZ (OR = 3.623, 95%CI[2.289–5.736], *P* = .000) were risk factors for ADRs. Some studies have been more in favor of pyrazinamide potential hepatotoxicity among the various components of a short-course anti-TB drug regimen.^[24]^ It was also reported that all first-line anti-TB drugs were associated with the development of ADRs, with Z being the most common offending drug, followed by S, E, R and H. Additionally, drug-induced hepatotoxicity was usually caused by H, R and Z, with Z being the most hepatotoxic and R being the least hepatotoxic.^[25]^ Since joint pain was caused by ethambutol-or pyrazinamide-induced hyperuricemia, patients who are prescribed these 2 drugs should have their serum uric acid levels measured.^[25]^ Therefore, these results implied that some pyrazinamide-containing regimens, such as HREZ, HRZS and HRZ, might be the risk factors for ADRs, but HRE might be more safer.

From Table 4, Type of TB (extrapulmonary, OR = 1.487, 95%CI[1.134–1.952]; *P* = .004) might be potential risk factor of ADRs. Notably, extrapulmonary TB (33.81%, 331/979) had higher incidence of ADRs than pulmonary TB (24.17%, 109/451). Therefore, these patents with extrapulmonary TB might be more likely to have ADRs during receiving HREZ, HRZS and HRZ regimens.

However, there are 2 main weaknesses of this study. Firstly, the study might be limited by its retrospective design. The available data was not enough or completely dependent on the medical records, and we could not follow up with the patients and manage ADRs during anti-TB therapy. Secondly, we did not collect patients’ information on performance status, bodyweight, patients’ education level, occupation, region, minority ethnic groups and nutritional status, which might be also risk factors for ADRs.

## 5. Conclusions

This study revealed that 30.77% of patients receiving anti-TB therapy developed one or more ADRs. Hyperuricemia was the most common ADR, followed by hepatic function test abnormality, liver damage and gastrointestinal reactions. Type of TB (extrapulmonary), treatment duration and therapeutic regimens were independently associated with ADRs during anti-TB therapy, respectively. These patents with extrapulmonary TB might be more likely to have ADRs during receiving HREZ, HRZS and HRZ regimens. Thus, for patients with these risk factors, it should be recommended to consider optimum management during anti-TB therapy, particularly hyperuricemia monitoring and hepatic function tests.

## Acknowledgments

We would like to express the warmest appreciation to all the staffs in the Department of Pharmacy, Renmin Hospital of Wuhan University.

## Author contributions

**Conceptualization:** Cai Shi, Boning Yang, Jingxiang Yang, Wei Song, Jian Yang.

**Data curation:** Cai Shi, Boning Yang, Jingxiang Yang, Wei Song, Ying Chen, Yuanguo Xiong, Peipei Rong, Yi Luo, Jian Yang.

**Formal analysis:** Cai Shi, Boning Yang, Jingxiang Yang, Wei Song, Shuxiao Zhang, Haiyan Zhan, Yuanguo Xiong, Peipei Rong, Yi Luo, Jian Yang.

**Funding acquisition:** Cai Shi, Ying Chen, Jian Yang.

**Investigation:** Cai Shi, Boning Yang, Jingxiang Yang, Wei Song, Jian Yang.

**Methodology:** Cai Shi, Boning Yang, Jingxiang Yang, Wei Song, Ying Chen, Shuxiao Zhang, Haiyan Zhan, Jian Yang.

**Project administration:** Cai Shi, Jingxiang Yang, Jian Yang.

**Resources:** Jian Yang.

**Software:** Jingxiang Yang, Jian Yang.

**Supervision:** Cai Shi, Jingxiang Yang, Jian Yang.

**Validation:** Cai Shi, Boning Yang, Jingxiang Yang, Wei Song, Jian Yang.

**Writing – original draft:** Cai Shi, Boning Yang, Jingxiang Yang, Wei Song, Ying Chen, Shuxiao Zhang, Haiyan Zhan, Yuanguo Xiong, Peipei Rong, Yi Luo, Jian Yang.

**Writing – review & editing:** Cai Shi, Boning Yang, Jingxiang Yang, Wei Song, Ying Chen, Shuxiao Zhang, Haiyan Zhan, Yuanguo Xiong, Peipei Rong, Yi Luo, Jian Yang.
